# Social bacteria and asocial eukaryotes

**DOI:** 10.1111/j.1462-2920.2007.01552.x

**Published:** 2008-02

**Authors:** Michael Y Galperin

**Affiliations:** National Center for Biotechnology Information, National Library of Medicine, National Institutes of HealthBethesda, MD 20894, USA

The end of 2007 brought us draft genome sequences of two eukaryotic microorganisms, *Babesia bovis* and *Malassezia globosa*, as well as complete genomes of the ammonia-oxidizing archaeal chemoautotroph *Nitrosopumilus maritimus*, and several environmental bacteria (Table 1). In terms of genome size, this list covers both sides of the spectrum: the 245 kb genome of an obligate insect symbiont *Sulcia muelleri* is the second smallest microbial genome sequenced so far, whereas the 13 033 kb chromosome of the social myxobacterium *Sorangium cellulosum* breaks the record as the largest bacterial genome – and the largest DNA molecule in the prokaryotic world.

**Table 1:** Recently completed microbial genomes (October–November 2007).

Species name	Taxonomy	GenBank accession	Genome size, bp	Proteins (total)	Sequencing centre^a^	Reference
**New organisms**						
*Babesia bovis*	*Eukaryota,Apicomplexa*	AACB02000000	8 200 000	6470	Washington State U.	Brayton *et al.* (2007)
*Malassezia globosa*	*Eukaryota,Basidiomycota*	AAYY00000000	8 900 000	4285	Procter & Gamble	Xu *et al.* (2007)
*Caldivirga maquilingensis*	*Crenarchaeota*	CP000852	2 077 567	1963	JGI	Unpublished
*Nitrosopumilus maritimus*	*Crenarchaeota*	CP000866	1 645 259	1798	JGI	Unpublished
*Frankia* sp. EAN1pec	*Actinobacteria*	CP000820	8 982 042	7191	JGI	Normand *et al*. (2007)
*Salinispora arenicola*	*Actinobacteria*	CP000850	5 786 361	4917	JGI	Unpublished
*Sulcia muelleri*	*Bacteroidetes*	CP000770	245 530	227	U. Arizona	McCutcheon and Moran (2007)
*Herpetosiphon aurantiacus*	*Chloroflexi*	CP000875, CP000876, CP000877	6 346 587 339 639 99 204	5278	JGI	Unpublished
*Acaryochloris marina*	*Cyanobacteria*	CP000828, CP000838–CP000846	8 361 599 (total)	8383	WashU	Unpublished
*Alkaliphilus oremlandii*	*Firmicutes*	CP000853	3 123 558	2836	JGI	Unpublished
*Clostridium phytofermentans*	*Firmicutes*	CP000885	4 847 594	3902	JGI	Unpublished
*Lactobacillus helveticus*	*Firmicutes*	CP000517	2 080 931	1625	Teagasc	Unpublished
*Azorhizobium caulinodans*	α-*Proteobacteria*	AP009384	5 369 772	4717	U. Tokyo	Unpublished
*Bartonella tribocorum*	α-*Proteobacteria*	AM260525, AM260524	2 619 061 23 343	2154	U. Basel	Saenz *et al.* (2007)
*Brucella canis*	α-*Proteobacteria*	CP000872, CP000873	2 105 969 1 206 800	3251	JGI, VBI	Unpublished
*Dinoroseobacter shibae*	α-*Proteobacteria*	CP000830–CP000835	1 159 772 total	1093	JGI	Unpublished
*Burkholderia multivorans*	α-*Proteobacteria*	CP000868–CP000871	7 008 622 total	6262	JGI	Unpublished
*Delftia acidovorans*	α-*Proteobacteria*	CP000884	6 767 514	6040	JGI	Unpublished
*Desulfococcus oleovorans*	δ-*Proteobacteria*	CP000859	3 944 167	3265	JGI	Unpublished
*Sorangium cellulosum*	δ-*Proteobacteria*	AM746676	13 033 779	9384	Bielefeld U.	Schneiker *et al.* (2007)
*Petrotoga mobilis*	*Thermotogae*	CP000879	2 169 548	1898	JGI	Unpublished
**New strains**						
*Methanococcus maripaludis* C6	*Euryarchaeota*	CP000867	1 744 193	1826	JGI	Unpublished
*Prochlorococcus marinus* str. MIT 9211	*Cyanobacteria*	CP000878	1 688 963	1855	JCVI	Unpublished
*Staphylococcus aureus* ssp. *aureus*USA300_TCH1516	*Firmicutes*	CP000730, CP000731, CP000256	2 872 915 27 041 3 125	2688	Baylor	Highlander *et al*. (2007)
*Salmonella enterica* ssp. *arizonae* serovar 62:z4,z23:--	γ*-Proteobacteria*	CP000880	4 600 800	4511	WashU	Unpublished
*Salmonella enterica* ssp. *enterica* serovar Paratyphi B str. SPB7	γ-*Proteobacteria*	CP000886	4 858 887	5601	WashU	Unpublished
*Shewanella baltica*OS195	γ-*Proteobacteria*	CP000891–CP000894	5 547 544 (total)	4688	JGI	Unpublished

a Sequencing centre names are abbreviated as follows: Baylor, Baylor College of Medicine, Houston, TX, USA; Bielefeld U., Center for Biotechnology, Bielefeld University, Bielefeld, Germany; JCVI, J. Craig Venter Institute, Rockville, MD, USA; JGI, US Department of Energy Joint Genome Institute, Walnut Creek, CA, USA; Procter & Gamble, The Procter and Gamble Co., Miami Valley Innovation Center, Cincinnati, OH, USA; Teagasc, Moorepark Food Research Centre, Moorepark, Fermoy, County Cork, Ireland; U. Basel, Focal Area Infection Biology, Biozentrum, University of Basel, Basel, Switzerland; U. Arizona, University of Arizona, Tucson, AZ, USA; U. Tokyo, University of Tokyo Biotechnology Research Center, Bunkyo-ku, Tokyo, Japan; VBI, Virginia Bioinformatics Institute at Virginia Polytechnic Institute and State University, Blacksburg, VA, USA; Washington State U., Washington State University, Pullman, WA, USA; WashU, Washington University Genome Sequencing Center, St Louis, MO, USA.

*Babesia bovis* is an apicomplexan parasite of cattle that is closely related to two other animal pathogens, *Theileria parva* and *Theileria annulata*, whose genomes have been sequenced in 2005. It is also related to such human pathogens as malaria-causing *Plasmodium* spp. and *Toxoplasma gondii*, the causative organism of human toxoplasmosis. Like *Plasmodium* spp., *B. bovis* infects and eventually lyses erythrocytes, causing anaemia. Studies of *B. bovis* infection, besides helping protect the livestock, could lead to a better understanding of malaria. The description of *B. bovis* genome sequence (Brayton *et al*., 2007) includes a detailed three-way comparison of the genomes and deduced protein sets of *B. bovis*, *Plasmodium falciparum* and *T. parva*. An interesting conclusion from this comparison is that the number of nuclear-encoded proteins targeted to the apicoplast (a plastid-like organelle of apicomplexans that is a potential target for antiparasite drugs) might be much smaller than previously believed. If true, this would have important consequences for future drug design. The knowledge of the genomic sequence will also boost the efforts towards creation of antibabesian vaccines.

Genomes of the basidiomycetes *Malassezia globosa* and *Malassezia restricta* are certain to attract attention of those who suffer from dandruff, a nasty skin flaking condition that reportedly occurs in 30–95% of the human population (Xu *et al*., 2007). These fungi also cause a more serious skin disease, referred to as seborrheic dermatitis. Analysis of *M. globosa* genome revealed a large number of secreted hydrolases (phospholipases, aspartyl proteases), but an apparent absence of a fatty acid synthase gene. This observation correlated with the experimental data on lipid requirement for growth of *M. globosa.* A somewhat unexpected result of the phylogenetic analysis of *M. globosa* proteins was that its nearest neighbour is apparently a plant pathogen, corn smut fungus *Ustilago maydis* (Xu *et al*., 2007).

Speaking about eukaryotic genomes, it might be appropriate to mention publication of the draft genome of the domestic cat *Felis catus* (Pontius *et al*., 2007). After all, this decidedly asocial eukaryote shares with us its home – and many microorganisms.

Compared with the tiny – for eukaryotic organisms – genomes of *B. bovis* and *M. globosa*, the genome of the soil myxobacterium *S. cellulosum* looks like a monster: at more than 13 Mbp, it is almost as large and encodes as many proteins as those two combined (Schneiker *et al*., 2007). Furthermore, it is composed of a single chromosome, in contrast to the genome of *B. bovis* that consists of four chromosomes of 2.62, 2.59, 1.73 and 1.25 Mbp in length (Brayton *et al*., 2007), and the genome of *M. globosa* that apparently consists of eight even smaller chromosomes (Xu *et al*., 2007). The chromosome of *S. cellulosum* turned out to be even longer than the initial estimate of 12.2 Mbp (Pradella *et al*., 2002) and is currently the largest DNA molecule known in the prokaryotic world, far surpassing the previous record, the 10 Mbp chromosome of *Solibacter usitatus*.

In the current classification, *S. cellulosum* belongs to the family *Polyangiaceae* in the order *Myxococcales* of the δ*-Proteobacteria*. *Myxococcus xanthus* and *Anaeromyxobacter dehalogenans*, whose genomes have been sequenced earlier (Goldman *et al*., 2006), belong to a different family, *Myxococcaceae*. *Stigmatella aurantiaca*, whose unfinished genome is available in GenBank (Accession No. AAMD00000000), is assigned to the family *Cystobacteraceae*. However, all these organisms are closely related and belong to the same order *Myxococcales* or ‘fruiting gliding bacteria’. The latter name captures three most prominent features of the group: (i) movement by gliding that includes ‘social’ and ‘adventurous’ motility, (ii) ability to form multicellular fruiting bodies with all the complex intercellular communication that is required for this process, and (iii) the fact that despite their large genome sizes, complex signalling and regulatory systems and a sophisticated social life, these organisms are still typical bacteria. The properties of myxobacteria, including *S. cellulosum,* have been subject of numerous reviews, including an opening paper in the inaugural issue of this journal (Reichenbach, 1999).

Unlike *M. xanthus* and *A. dehalogenans* that are remarkable for their predatory lifestyle and aryl-halorespiration, respectively, *S. cellulosum* has attracted attention primarily by its ability to degrade cellulose and produce a great variety of secondary metabolites with antibacterial and antifungal activity. This organism was first described as *Polyangium compositum* in 1904, renamed *Sorangium compositum* in 1924 and received its current name in 1936 (Imshenetski and Solntseva, 1937). The sequenced strain *S. cellulosum* So ce56 was isolated in 1985 in Germany from a soil sample containing decaying plant material, which had been collected near Cipajung in Indonesia. Genome analysis of *S. cellulosum* revealed more than 3000 proteins (34.7% of the total) that had no significant similarity to predicted proteins in the public databases (Schneiker *et al*., 2007). Efforts to deduce their functions through genomic context-based methods, including so-called phylogenomic maps, also proved unsuccessful, making *S. cellulosum* the largest source of unannotated bacterial proteins. A comparison of *S. cellulosum* and *M. xanthus* genomes revealed an almost complete lack of synteny. Like *M. xanthus*, *S. cellulosum* encoded a very complex system of signal transduction proteins that included more than 140 histidine kinases and more than 300 eukaryotic-type serine/threonine/tyrosine protein kinases, comprising more than 3% of all predicted proteins (Schneiker *et al*., 2007). In accordance with its lifestyle, *S. cellulosum* encoded only five chemotaxis transducers (MCPs), four times less than *M. xanthus*, but had a larger fraction of genes involved in carbohydrate metabolism. A significant part of the analysis was dedicated to the enzymes of secondary metabolism, particularly polyketide synthases and non-ribosomal peptide synthases. The genes responsible for synthesis of chivosazol, etnangien and myxochelin have been identified, and several more gene clusters encoding polyketide synthase and/or non-ribosomal peptide synthase domains have been found in the genome. Characterization of these genes could result in discovery of entirely new antibiotics. In addition, comparative analysis of signalling systems of *M. xanthus* and *S. cellulosum* could shed light on the mechanism of their social behaviour.

The list of newly sequenced genomes (Table 1) includes two members of the *Crenarchaeaota*. *Caldivirga maquilingensis* is a hyperthermoacidophile, originally isolated from a hot spring located on the side of Mount Maquiling, Laguna, in the Philippines (Itoh *et al*., 1999). It grows in a wide range of temperature and pH values with optimal growth at 85°C and pH around 4.0. This organism is a heterotroph that can utilize gelatin, peptone and other protein substrates as carbon sources. It can grow both anaerobically and microaerobically, but requires sulfur, thiosulfate or sulfate as electron acceptors. *Caldivirga maquilingensis* forms a separate branch in the family *Thermoproteaceae*, which also includes the genus *Pyrobaculum* with its four completed genomes. Comparison of *C. maquilingensis* genome with those from *Pyrobaculum* spp. is expected to shed light on the mechanisms of aerotolerance in hyperthermophilic archaea and their choice of terminal electron acceptors.

*Nitrosopumilus maritimus* strain SCM1 was isolated in 2005 from a marine tropical fish tank at the Seattle aquarium and was the first cultivated non-thermophilic crenarchaeon, a representative of the vast community of crenarchaea inhabiting cold oxic ocean waters (Könneke *et al*., 2005). In addition, *N. maritimus* was the first ammonia-oxidizing archaeal chemoautotroph obtained in pure culture. Its ability to use bicarbonate and ammonia as sole sources of carbon and energy is apparently widespread in the open ocean (Ingalls *et al*., 2006; Coolen *et al*., 2007).

The two members of the phylum *Actinobacteria* in the current list illustrate the diversity of the group. The soil nitrogen-fixing symbiotic actinobacterium *Frankia* sp. strain EAN1pec is the third representative of that genus with a completely sequenced genome. All three genomes were sequenced in 2006 in the course of a large Franco-American project and described in a paper (Normand *et al*., 2007) that is already freely available online. However, genome of the strain EAN1pec, the largest of the three, included some gaps corresponding to regions with sequence repeats and high GC content. These gaps have now been filled and the genome has been released in the finished form. More information on *Frankia* and actinorhizal plants is available at http://web.uconn.edu/mcbstaff/benson/Frankia/FrankiaHome.htm

The second actinobacterium in the list is *Salinispora arenicola*, a marine organism that is found in tropical and subtropical marine sediments around the world at the depth of up to 1100 m (Mincer *et al*., 2005; Jensen and Mafnas, 2006). The genus *Salinispora* includes numerous marine isolates but only one other validly described species, *Salinispora tropica* (Maldonado *et al*., 2005), whose genome was released by the JGI 6 months earlier (Udwary *et al*., 2007). Genomes of two more strains, one from *S. arenicola* and one from *S. tropica*, are currently in the works. *Salinispora* spp. grow only in the presence of seawater or on sodium-enriched media (Mincer *et al*., 2002; Maldonado *et al*., 2005), which makes them obligate marine bacteria and points to a very interesting evolutionary history. Another reason for sequencing *Salinispora* spp. is their complex secondary metabolism. Many *Salinispora* isolates produce biologically active compounds that inhibit proliferation of tumour cells and are promising candidates for anticancer therapy. These include rifamycin-like compounds, cyclopenta[a]indene glycosides and halogenated macrolides. One of such compounds, salinosporamide A (Protein Data Bank entry 2FAK, see http://pubchem.ncbi.nlm.nih.gov/summary/summary.cgi?sid=11110244 for a chemical formula), is a potent inhibitor of the 20S proteasome that is currently in phase I clinical trials for the treatment of cancer. *Salinispora arenicola* produces various macrolide polyketides, such as saliniketals A and B (inhibitors of ornithine decarboxylase) and arenicolides A, B and C (Williams *et al*., 2007a,b), whose biosynthetic pathways will now be investigated using the genomic sequence.

*Sulcia muelleri* was described in 2005 as an obligate bacterial symbiont of the glassy-winged sharpshooter (*Homalodisca coagulata*), a 12-mm-long leafhopper that feeds on xylem fluid of a wide range of plants (Moran *et al*., 2005). Unlike previously known obligate insect symbionts, it is not a γ-proteobacterium but a member of the phylum *Bacteroidetes* that functions as a co-symbiont with the γ-proteobacterium *Baumannia cicadellinicola*. The complete genome sequence of *B. cicadellinicola* and partial genome sequence of *S. muelleri* have been determined, leading to the suggestion that these two bacteria have complementary metabolic capabilities (Wu *et al*., 2006). The genome sequence of *S. muelleri* has now been completed, using a combination of pyrosequencing with even shorter reads of 33 bases in length generated by an Illumina/Solexa Genome Analyzer (McCutcheon and Moran, 2007). Genome sequence of *S. muelleri* made it possible to analyse its metabolism in detail and verify the idea of the metabolic interdependence of *S. muelleri*, *B. cicadellinicola* and the sharpshooters. Indeed, *S. muelleri* was found to encode biosynthetic pathways for all essential amino acids, but not purines, pyrimidines, vitamins and cofactors, whereas *B. cicadellinicola* can produce vitamins and cofactors, purines and pyrimidines but not amino acids and the host cells cannot produce either of these. These shared functions ensure that neither cell can exist without the others and force them all to co-evolve (McCutcheon and Moran, 2007). Given that intracellular bacterial symbionts from the *Bacteroidetes* lineage were also found in cicadas, leafhoppers, treehoppers, spittlebugs and planthoppers (Moran *et al*., 2005), such tripartite symbioses may be widespread in nature.

*Herpetosiphon aurantiacus* was first isolated in 1961 from the slimy coating of *Chara* sp. growing in Birch Lake in Minnesota and described as a separate organism in 1968, based on isolation of similar strains from well water and cow dung in Iowa, hot springs in California and Mexico and from marine shores of France, Eire, Lagos and Samoa (Holt and Lewin, 1968). Closely related strains were later isolated from bulking activated sludge at communal and industrial sewage treatment plants in southern Germany (Trick and Lingens, 1984). *Herpetosiphon aurantiacus* is a Gram-negative aerobic filamentous gliding bacterium that belongs to the phylum *Chloroflexi*, also referred to as *green non-sulfur bacteria*, or *GNS group*, and has an unusual cell wall, typical for those organisms. The cells divide by transverse septum formation, forming long flexible filaments that consist of numerous cells and can be 0.2–0.5 mm in length. Unlike its relatives, such as *Chloroflexus aurantiacus* and *Roseiflexus castenholzii*, *H. aurantiacus* does not produce bacteriochlorophyll and is unable to perform anoxygenic photosynthesis. The genome sequence should provide insights into the specific growth patterns of *H. aurantiacus*, as well as into its unusual ability to kill a wide variety of bacteria by lysing their colonies (Quinn and Skerman, 1980) and to produce unique secondary metabolites (Nett *et al*., 2006).

*Acaryochloris marina* is a relatively well-studied model organism that was first isolated in 1993 from algae squeezed out of *Lissoclinum patella*, a colonial ascidian collected from the marine coast of the Palau Islands in the western Pacific Ocean (Miyashita *et al*., 1996). It had an unusual morphology and an unusual photosynthetic pigment, chlorophyll *d*, seen previously only in red algae. However, 16S rRNA analysis clearly showed that *A. marina* is a member of *Cyanobacteria* (Miyashita *et al*., 2003). Furthermore, chlorophyll *d* of red algae was shown to be produced by *A. marina*-like bacteria, associated with algal cells (Murakami *et al*., 2004). Spectral properties of chlorophyll *d* allow *A. marina* to use far-red light for photosynthesis, defining its unique ecological niche underneath the coral-reef ascidians (Kühl *et al*., 2005).

Among the four low-G+C Gram-positive bacteria in the current list, there are two interesting representatives of the family *Clostridiaceae*. One of them, *Alkaliphilus oremlandii* strain OhILAs, is yet another example of a bacterium whose complete genome sequence had been released even before the organism was formally described. This bacterium was isolated from sediments of the Ohio River near Pittsburgh, Pennsylvania, using a medium with 10 mM arsenate and 20 mM lactate, and was originally referred to as *Clostridium* sp. OhILAs (Fisher *et al*., 2008). The organism used arsenate or thiosulfate as terminal electron acceptors and tolerated up to 50 mM of arsenate. Accordingly, it was named *Clostridium oremlandii* after Ronald S. Oremland, an investigator with the US Geological Survey and a member of the Editorial Board of this journal, who had been studying bacterial use of arsenate for many years (Oremland and Stolz, 2003; 2005). Finally, 16S rRNA sequence clearly identified this bacterium as a member of the genus *Alkaliphilus*, despite its ability to grow only in a relatively narrow pH range of 8.0–8.8. About a year ago, *A. oremlandii* attracted attention of the general public with its ability to degrade 3-amino-4-hydroxybenzene arsonic acid (trade name: Roxarsone, see http://pubchem.ncbi.nlm.nih.gov/summary/summary.cgi?cid=5104), a compound that is added to the broiler chicken feed to prevent coccidiosis (an infection by the apicomplexan protist *Eimeria* spp.), but also stimulates chicken growth and improves pigmentation (Stolz *et al*., 2007). In these experiments, most of the roxarsone ended up reduced to 3-amino-4-hydroxybenzene arsonic acid, but a significant fraction was apparently degraded further releasing inorganic arsenic, a well-known poison and a human carcinogen. The idea that roxarsone, which has been abandoned in Europe since 1999 but is still being added to the feed of ∼70% of broilers in the USA, could end up contaminating soil and groundwater with arsenate is quite unsettling. Several major firms have stopped using roxarsone or pledged to do that, but the continued use of organoarsenic compounds and the ability of clostridia to degrade them are certain to remain a subject of much controversy.

The second *Clostridium*, *C. phytofermentans*, is an obligately anaerobic mesophilic cellulolytic bacterium, isolated from forest soil in central Massachusetts. It is capable of fermenting a variety of complex carbohydrates, including cellulose, pectin, polygalacturonic acid, starch and xylan, to ethanol, acetate, CO_2_ and H_2_ (Warnick *et al*., 2002). Production of ethanol as the major end-product of fermentation makes *C. phytofermentans* an attractive organism for biofuel production. A comparison of its genomic sequence with that of *Clostridium thermocellum*, released by the JGI a year ago, should help understand its peculiar metabolism and increase ethanol production by other cellulolytic bacteria.

*Lactobacillus helveticus*, first described in 1919 by Orla-Jensen, is a moderately thermophilic lactic acid bacterium, used in production of cheese. It belongs to the *Lactobacillus acidophilus* subgroup of lactobacilli and is characterized by a diminished capacity to ferment sugars. *Lactobacillus helveticus* is primarily used in starter cultures in the manufacture of Swiss-type and long-ripened Italian cheeses, such as Emmental, Gruyère and Provolone. The sequenced strain DPC 4571 is a Swiss cheese isolate that has been selected for a number of highly desirable traits including rapid autolysis, reduced bitterness and increased flavour notes (Hickey *et al*., 2007). The genome sequence of *L. helveticus* revealed a high level of synteny with *L. acidophilus*, numerous IS elements and an apparent loss of genes that contribute to the colonization of the intestinal mucosa in probiotic lactobacilli (Callanan *et al*., 2008).

Two of the four α-proteobacterial genomes in the current list come from obligate parasites: *Bartonella tribocorum* is an intraerythrocytic pathogen of rats, while *Brucella canis*, as the name suggests, is the causative agent of canine brucellosis. The other two represent interesting environmental microorganisms. The nitrogen-fixing bacterium *Azorhizobium caulinodans* strain ORS571 is a member of so-called fast-growing group of rhizobia. This organism was first isolated in 1981 from nitrogen-fixing nodules formed on the stem of the tropical legume *Sesbania rostrata*, commonly found in freshwater swamps in Africa, by the scientists from the Laboratoire de Microbiologie des Sols, Office de la Recherche Scientifique et Technique Outre-Mer in Dakar, Senegal (Dreyfus and Dommergues, 1981), and could be counted among the greatest discoveries ever made by that office. *Azorhizobium caulinodans* could grow in pure culture using N_2_ as the sole nitrogen source and, unlike other rhizobia, readily nodulated legume stems (Elmerich *et al*., 1982; Dreyfus *et al*., 1983), offering a convenient experimental model to study mechanisms of nodulation and nitrogen fixation. In the past 20 years, *A. caulinodans* has been used as model organism in numerous studies. One of such studies revealed that, in addition to forming nodules, this bacterium could colonize xylem of *S. rostrata* roots (O'Callaghan *et al*., 1997). Colonization of root xylem by *A. caulinodans* was subsequently shown for *Arabidopsis*, rice and tomato, suggesting that root xylem could provide a suitable niche for endophytic nitrogen fixation (Gopalaswamy *et al*., 2000; Stone *et al*., 2001). Recently, a transposon mutagenesis study of the mechanisms of the maturation and maintenance of N_2_-fixing nodules identified novel symbiosis-related genes in *A. caulinodans* (Suzuki *et al*., 2007). This must have prompted sequencing of its genome, which will now further boost the use of *A. caulinodans* as a model organism. Stem nodulation, bacterial colonization of xylem and nodulation in the absence of *nod* genes (Giraud *et al*., 2007) show that bacteria-plant symbiosis is even more complex than we used to think.

The key properties of another environmental α-proteobacterium, *Dinoroseobacter shibae*, are perfectly reflected in its name. The genus name identifies it as a member of the marine *Roseobacter* clade; the first two syllables reflect its isolation from cultivated marine dinoflagellates. The species name has been assigned after Professor Tsuneo Shiba of the University of Tokyo Otsuchi Marine Research Centre, who discovered the marine aerobic anoxygenic phototrophs and provided the first description of this important group of bacteria (Shiba *et al*., 1979). Cultures of *D. shibae* grown in the dark accumulate the carotenoid pigment spheroidenone (http://pubchem.ncbi.nlm.nih.gov/summary/summary.cgi?cid=5366412) and are characterized by intense colour, from pink to wine-red (Biebl *et al*., 2005). They also contain bacteriochlorophyll *a* and are able to perform aerobic anoxygenic photosynthesis. Still, *D. shibae* is a heterotroph that can use acetate, succinate, fumarate, malate, lactate, citrate, glutamate, pyruvate, glucose, fructose and glycerol as carbon sources. The genome sequence of *D. shibae* should allow further analysis of these poorly studied microorganisms.

The soil bacterium *Delftia acidovorans* has been first described in the PhD thesis of L.E. den Dooren de Jong (1926) with ∼40 other *Pseudomonas* soil isolates and named *Pseudomonas acidovorans*. Very similar strains have been described as *Pseudomonas desmolytica, Pseudomonas indoloxidans* and *Pseudomonas testosteroni*. These strains were subsequently re-classified as β-proteobacteria and combined under the name *Comamonas acidovorans*. Finally, in 1999, this organism received its current name, ‘referring to the city of Delft, the site of isolation of the type species, and in recognition of the pioneering role of Delft research groups in the development of bacteriology’ (Wen *et al*., 1999). Representatives of this species have been found in a variety of environments, including soil, river sediment, activated sludge, wastewater and even drinking water. They have extremely versatile metabolism and degrade a wide variety of pollutants. The sequenced strain *D. acidovorans* DSM 14801 was isolated at a communal sewage treatment plant in Germany and shown to utilize 4-(4-sulfophenyl)-hexanoate (Schleheck *et al*., 2004). This and other strains of *D. acidovorans* are attractive candidates for use in bioremediation.

The two remaining bacteria in the current list are both obligate anaerobes isolated from oil production plants. *Desulfococcus oleovorans* is a sulfate-reducing δ-proteobacterium that was isolated using hexadecane as the sole carbon source. This organism could grow on alkanes from C_12_ to C_20_, 1-hexadecene, 1-hexadecanol, 2-hexadecanol, palmitate and stearate using sulfate as terminal electron acceptor (Aeckersberg *et al*., 1991). It has been proposed that reduction of sulfate in oil by *D. oleovorans* and related bacteria could be responsible for the accumulation of sulfide in oil deposits and oil production plants (Rueter *et al*., 1994).

*Petrotoga mobilis* is a moderately thermophilic member of the phylum *Thermotogae.* This bacterium was isolated from hot production waters of a North Sea oil reservoir and grows optimally at 58–60°C (Lien *et al*., 1998). The JGI *Thermotogales* project sequenced the genome of *P. mobilis* primarily for comparing it with the genomes of *Fervidobacterium nodosum* and *Thermosipho melanesiensis*, whose optimal growth temperature is 10°C higher, and *Thermotoga maritima* and *Thermotoga petrophila*, whose optimal growth temperature is 20°C higher. Such a comparison could provide valuable clues to the hyperthermophilic adaptations within this ancient clade.

Several other recently sequenced genomes come from important pathogens. *Burkholderia multivorans* is a member of the *Burkholderia cepacia* complex, an opportunistic pathogen that colonizes lungs of patients with cystic fibrosis, aggravating the condition and sometimes causing fatal necrotizing pneumonia.

Two new strains of *Salmonella enterica* have been sequenced in the course of the Enterobacterial genome project at the Washington University School of Medicine (http://genome.wustl.edu/sub_genome_group.cgi?GROUP=3&SUB_GROUP=3). *Salmonella enterica* ssp. IIIa (arizonae) serovar 62:z4,z23:-- (common name: *Salmonella* Arizonae) is a reptile isolate that can infect turkeys and occasionally causes gastroenteritis in humans, while *S. enterica* ssp. *enterica* serovar Paratyphi B str. SPB7 (common name: *Salmonella* Paratyphi B) infects only humans, causing a typhoid-type fever.

Methicillin-resistant *Staphylococcus aureus* (MRSA) is an important pathogen that is spreading around the world. To get a better understanding of this growing threat, researchers at the Baylor College of Medicine sequenced genomes of two paediatric isolates of *S. aureus* strain USA300, one of the most virulent strains that causes superficial and invasive infections in children and adults (Highlander *et al*., 2007). A comparison of methicillin-resistant and methicillin-sensitive isolates did not reveal any major differences in gene order and plasmid content, indicating that the differences in virulence of these strains are not due to acquisition of specific virulence factors. The authors suggest that pathogenicity differences between *S. aureus* strains are largely determined by sequence polymorphisms (Highlander *et al*., 2007).

Finally, there has been an interesting development in the area of genome annotation. For a number of years, function of one of the most widespread ‘conserved hypothetical’ genes remained ambiguous. This gene, referred to as *ygjD* (*gcp*) in *Escherichia coli*, *ydiE* in *Bacillus subtilis*, QRI7 and Kae1 in yeast, and OSGEP and OSGEPL1 in humans, is one of the very few to be encoded in every sequenced genome (with the exception of highly degraded genomes of *Carsonella ruddii* and, now, *S. muelleri*). Until recently, the only key to its function was an experimental paper showing that in *Pasteurella haemolytica* A1, the product of this gene was a glycoprotease, specific for O-sialoglycoproteins (Abdullah *et al*., 1991), which is how this gene is currently annotated in most public databases. That function did not seem appropriate for such a widespread gene, which prompted us to place it as No. 1 in our ‘top 10’ list of ‘known unknown’ proteins that should be priority targets for further experimental study (Galperin and Koonin, 2004). In a recent paper in *Nucleic Acids Research*, a group of French scientists led by Patrick Forterre reported the absence of protease activity in the archaeal (*Pyrococcus abyssi*) orthologue of this protein. Instead, the expressed protein could bind DNA and exhibited an apurinic endonuclease activity (Hecker *et al*., 2007). It still was not immediately clear what biological role would require presence of this protein in every organism on this planet, but interaction with DNA sounds far more plausible than hydrolysis of glycoproteins.
